# The role of artificial intelligence and machine learning in harmonization of high-resolution post-mortem MRI (virtopsy) with respect to brain microstructure

**DOI:** 10.1186/s40708-019-0096-3

**Published:** 2019-03-07

**Authors:** Shane O’Sullivan, Helmut Heinsen, Lea Tenenholz Grinberg, Leila Chimelli, Edson Amaro, Paulo Hilário do Nascimento Saldiva, Fleur Jeanquartier, Claire Jean-Quartier, Maria da Graça Morais Martin, Mohammed Imran Sajid, Andreas Holzinger

**Affiliations:** 1 Department of Pathology, Faculdade de Medicina, Universidade de Sao Paulo, São Paulo, Brazil; 2 Morphological Brain Research Unit, University of Würzburg, Würzburg, Germany; 3 Institute of Radiology, Faculdade de Medicina, Universidade de Sao Paulo, São Paulo, Brazil; 4 Aging Brain Project, Department of Pathology, Faculdade de Medicina, Universidade de Sao Paulo, São Paulo, Brazil; 5 Albert Einstein Instituto Israelita de Ensino e Pesquisa, São Paulo, Brazil; 6 Laboratory of Neuropathology, State Institute of Brain, Rio de Janeiro, Brazil; 7 Institute of Advanced Studies, Universidade de Sao Paulo, São Paulo, Brazil; 8 Holzinger Group, Institute for Medical Informatics and Statistics, Medical University of Graz, Graz, Austria; 9 Department of Upper GI Surgery, Wirral University Teaching Hospital, Birkenhead, United Kingdom

**Keywords:** Neuroimaging, Neurodegenerative diseases, Stereology, Brain mapping, Disector, 7 T post-mortem MRI

## Abstract

Enhanced resolution of 7 T magnetic resonance imaging (MRI) scanners has considerably advanced our knowledge of structure and function in human and animal brains. Post-industrialized countries are particularly prone to an ever-increasing number of ageing individuals and ageing-associated neurodegenerative diseases. Neurodegenerative diseases are associated with volume loss in the affected brain. MRI diagnoses and monitoring of subtle volume changes in the ageing/diseased brains have the potential to become standard diagnostic tools. Even with the superior resolution of 7 T MRI scanners, the microstructural changes comprising cell types, cell numbers, and cellular processes, are still undetectable. Knowledge of origin, nature, and progression for microstructural changes are necessary to understand pathogenetic stages in the relentless neurodegenerative diseases, as well as to develop therapeutic tools that delay or stop neurodegenerative processes at their earliest stage. We illustrate the gap in resolution by comparing the identical regions of the post-mortem in situ 7 T MR images (virtual autopsy or virtopsy) with the histological observations in serial sections through the same brain. We also described the protocols and limitations associated with these comparisons, as well as the necessity of supercomputers and data management for “Big data”. Analysis of neuron and/or glial number by using a body of mathematical tools and guidelines (stereology) is time-consuming, cumbersome, and still restricted to trained human investigators. Development of tools based on machine learning (ML) and artificial intelligence (AI) could considerably accelerate studies on localization, onset, and progression of neuron loss. Finally, these observations could disentangle the mechanisms of volume loss into stages of reversible atrophy and/or irreversible fatal cell death. This AI- and ML-based cooperation between virtopsy and histology could bridge the present gap between virtual reality and neuropathology. It could also culminate in the creation of an imaging-associated comprehensive database. This database would include genetic, clinical, epidemiological, and technical aspects that could help to alleviate or even stop the adverse effects of neurodegenerative diseases on affected individuals, their families, and society.

## Introduction

In the past two centuries, post-mortem autopsy and histopathological investigation of diseased organs or tissues have yielded innumerable observations on disease onset, progression, and fatal outcome. Knowledge of the pathogenesis of diseases proved indispensable for disease diagnosis, therapy, and prevention. Ethical issues including retention of organs after autopsy [1] and financial considerations [2] may have contributed to a global decrease in autopsy rates [3–11]. Virtual autopsy (Virtopsy) [12], in the form of MRI or computed tomography (CT) post-mortem investigation, could reduce the financial and physical/psychological burden [4, 5, 13–19] of autopsies, as well as appease fears and increase the autopsy rate amongst large populations of individuals with strong traditional, religious or cultural concerns [20]. Previous reviews still considered both, autopsy and virtopsy, supplementing each other [21, 22].

The introduction of 7 T high-resolution MRI-scanners with superior resolution resulted in more detailed images of the human central nervous system, which is characterized by densely packed neurons, neurites, glial cells, and a typical vascular supply of cortical and subcortical regions. High-detailed 3D reconstructions of basal ganglia elements and circuits [23–25] may help in targeting defined nuclear regions for neurostimulation in the course of Parkinson’s disease, and prevent or reduce unwanted side effects. We present unpublished exemplary observations of a combined 3 T in situ, 7 T post-mortem MRI investigation compared with horizontal serial 420-μm-thick gallocyanin-stained sections of the complete brain of an 85-year-old female [26–28].

Our neuropathological data illustrate the complexity involved with tasks that aim to identify many different cerebral disorder patterns. The study was conducted in cooperation with the Autopsy Service (SVO) of the University of Sao Paulo (USP). We describe the current status of virtopsy and provide practical examples demonstrating how this consists of two stages: early post-mortem MRI of the brain, followed by taking biopsies at selected brain regions guided by the findings of the MRI. Finally, we provide guidelines on how MRI analysis can be enhanced by artificial intelligence (AI) and machine learning (ML). We argue that AI and ML can effectively help with challenges highlighted in this paper, and also identify many other benefits (such as more accurate identifications and faster results among other benefits).

## A comparison between post-mortem in situ 3 T and 7 T MR imaging after autopsy with formalin fixation and a gallocyanin-stained celloidin section

**Fig. 1:**
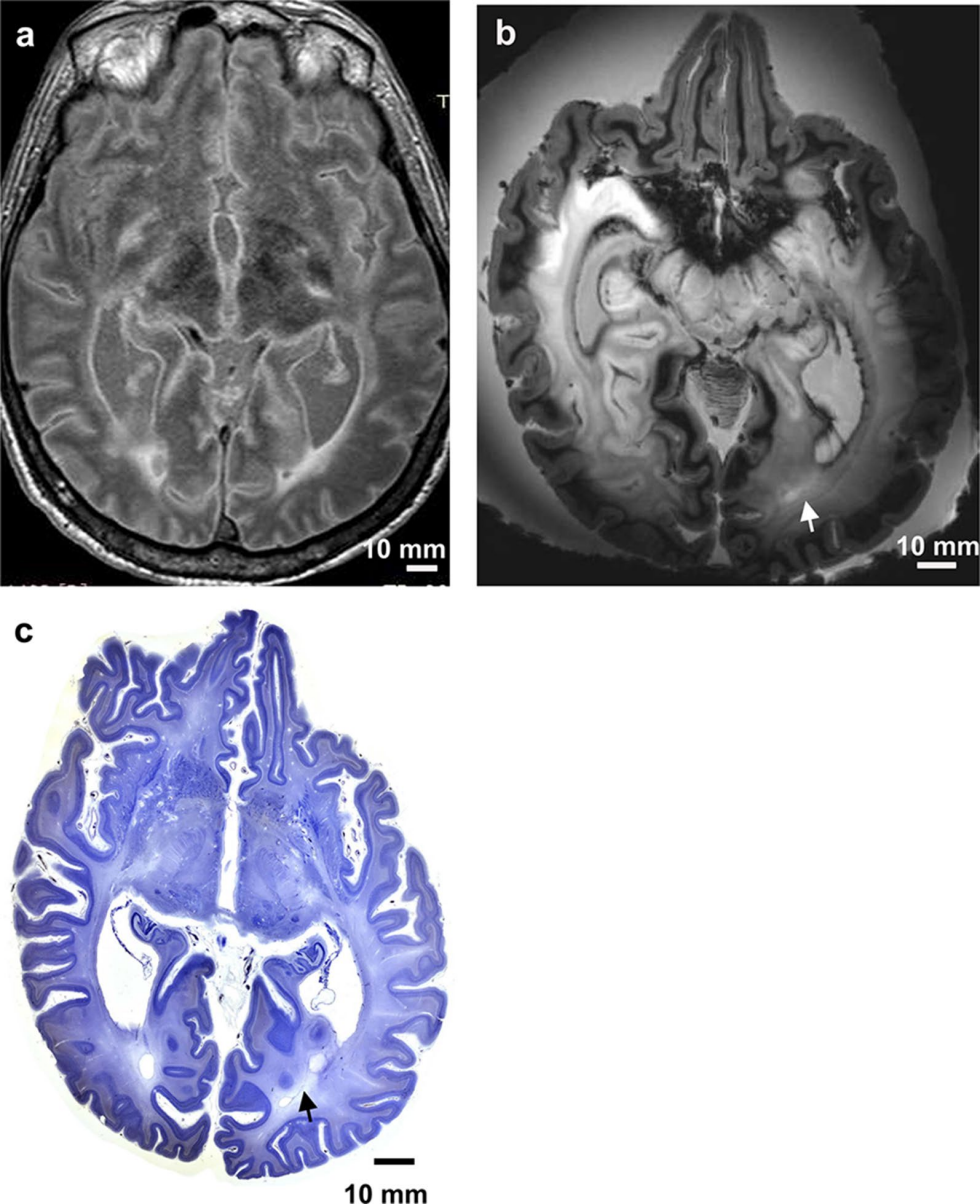
Panel of 3 Tesla MRI versus 7 Tesla MRI: this panel includes a comparison of a 7 Tesla MRI versus a 3 Tesla MRI for the same brain. **a** 3 Tesla axial FLAIR MR image of a horizontally cut brain, thickness = 2.0 mm, and unfixed post-mortem in situ. **b** Same brain as in **a** after removal from the cranial cavity and formalin fixation for more than 3 months. 7 Tesla axial T2 GRE MR image (TE = 21 s, TR = 25 s) and thickness = 1.0 mm. **c** Histological serial section through a human brain, celloidin embedding, section thickness = 420 μm, and gallocyanin staining. This histology picture was taken by a Nikon D800E SLR camera with a Sigma 2.8/50 mm macrolens. This is an example of how histology data can supplement 7 Tesla MRI data. Scalebar = 10 mm and is the same for all three images. Arrows in **b** and **c** point to white matter hyperintensities

The 3 T in situ image taken at a post-mortem time of 16 h depicts a well-preserved brain with a high contrast between cortex, medullary layer, and subcortical nuclei (Fig. 1a).

**Fig. 2:**
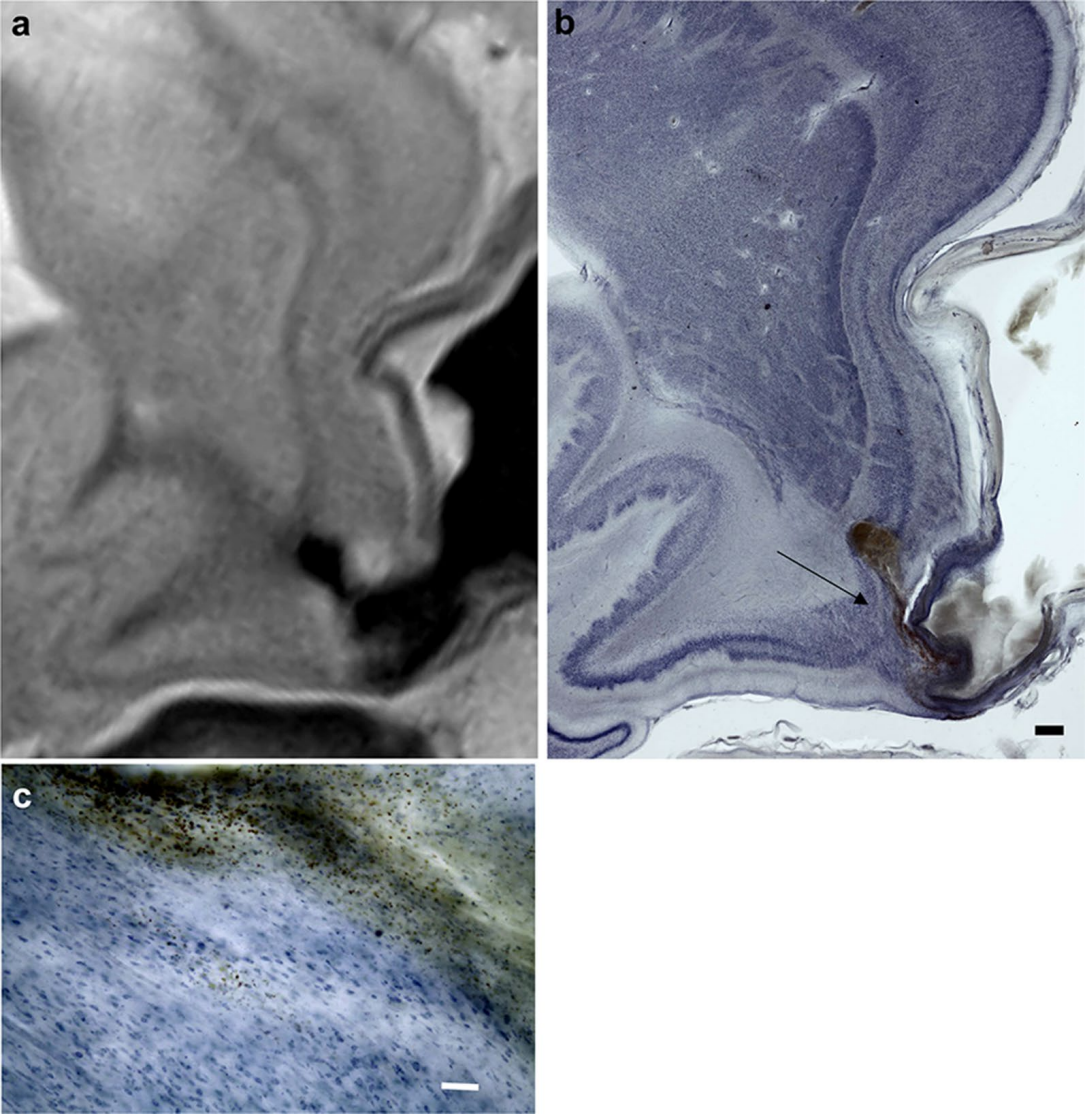
Horizontally cut right parahippocampal gyrus of an 85-year-old female with aneurysm of the right medial cerebral artery. **a** 7 T scan of the formalin-fixed brain. **b** Macroscopic overview of a gallocyanin-stained 420-μm-thick celloidin section through the corresponding region as depicted in **a**. **c** Microscopic photograph of the region indicated by the arrow in **b** with the majority of neurons unimpaired even when close to the fountain-like streak of hemosiderin emerging from the caudo-lateral extreme of the aneurysm. Scalebar in **b** = 1 mm and in **c** it is = 0.1 mm

An in situ post-mortem image is indispensable for comparison of either 3 T or 7 T MR images and histology. Figure 1b documents the same brain after formalin fixation. In general, the resolution of a 7 T scanner is far superior to a 3 T scanner. However, fixation artefacts by formalin cause a nutshell-phenomenon (Fig. 2b). Initially, formalin rapidly penetrates human central nervous tissue. After a few millimetres fixation-induced cross-linking of proteins imposes a kind of barrier that impedes a further penetration of formalin into the deepest parts of the brain. Early opening of the telencephalic ventricular system by a sagittal cut into the corpus callosum facilitates the replacement of cerebrospinal fluid (CSF) by formalin with a concomitant fixation of external and internal brain parts.

Care must be taken during the first 12 h of fixation because incomplete fixed brain tissue is soft and subject to considerable and irreversible distortion. Intracranial perfusion fixation via the carotid and basilar arteries would be an optimal strategy for brain fixation without distortion. However, this strategy is usually only possible with brains from patients who were enrolled in body donation programs of anatomical institutes. Perfusion fixation does not guarantee the absence of fixation artefacts because post-mortem blood clotting and frequently encountered arteriosclerotic plaques in elderly individuals can impede the complete removal of blood from the brain’s vascular system.

Moreover, high-pressure intravascular injection of formalin can cause rupture of cerebral arteries and mechanic fixation artifacts. Immediately after the removal from the skull, unfixed brains should be weighed and carefully suspended into an excess of low concentration formalin (one part with 37% formaldehyde and 9 parts with distilled water—at least 5 liters) with a small thread on the walls of a fixation bucket. Incomplete fixation and storage of the brain on the base of a bucket instead of suspension could explain the distortion of the brain (compare Fig. 1a with b). Neurons, a major component of the central nervous system, can only be visualized after cutting of the brain and staining with special stains that are summarized under the term Nissl stains. Prior to cutting, the brain must be dehydrated with alcohol and embedded into either paraffin or celloidin. This procedure causes considerable shrinkage of the brain with a volume loss of up to 60%. Different shrinkages of the brain’s white and grey matter during dehydration and embedding are additional confounders causing further brain deformation [12].

**Fig. 3:**
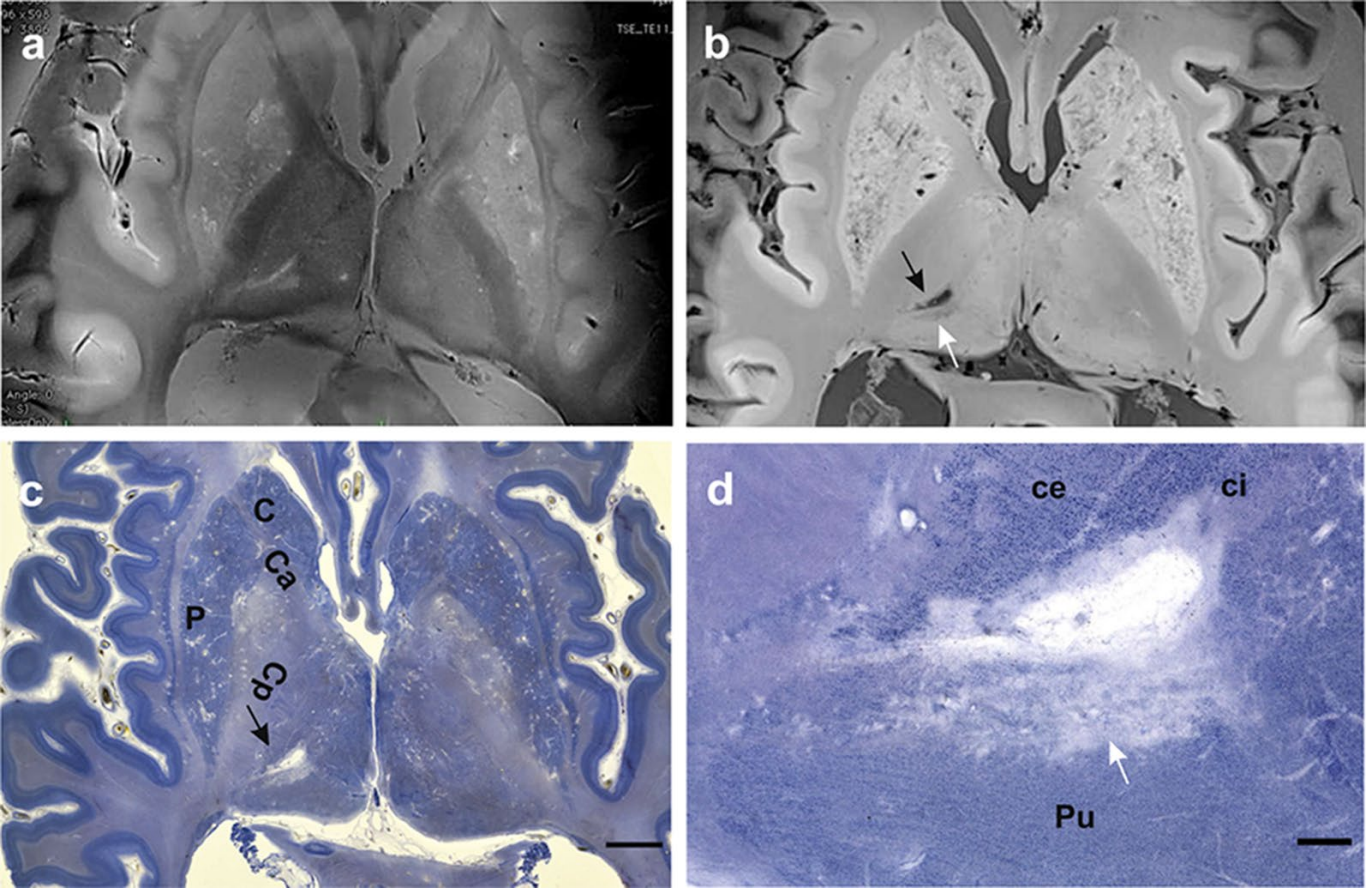
**a** 7 T MRI of a post-mortem fixated brain. T2 TSE axial image, 2.0 mm thickness and 0.2 mm in plane resolution. **b** 7 T MRI of a post-mortem fixated brain. 3D GRE TR 12 s, TE 8 s, 0.3 mm in plane resolution and 0.6 mm slice thickness. Black arrows in **b** and **c** point to a small liquifying infarct in the posterior region of the left thalamus. **c** Celloidin section of the same brain as depicted in **a** and **b**. 420 μm section thickness and gallocyanin staining. **d** Higher magnification of the region with small liquified infarct at the border of the posterior ventrocaudal nucleus and the pulvinar thalami. The shape and the size of the infarct region are similar after 7 T imaging and after celloidin embedding of the brain that was cut into 283 horizontal sections. The scalebar in **c** = 5 mm long and in **d** it is = 1 mm width. **c** and **d** show C = caudate nucleus, Ca = crus anterius, Cp = crus posterius of the internal capsule, ce = external part, ci = internal part of the thalamic ventrocaudal nucleus, Pu = pulvinar thalami, and P = putamen. White arrows in **b** and **d** point to a region of elective parenchymal necrosis

**Fig. 4:**
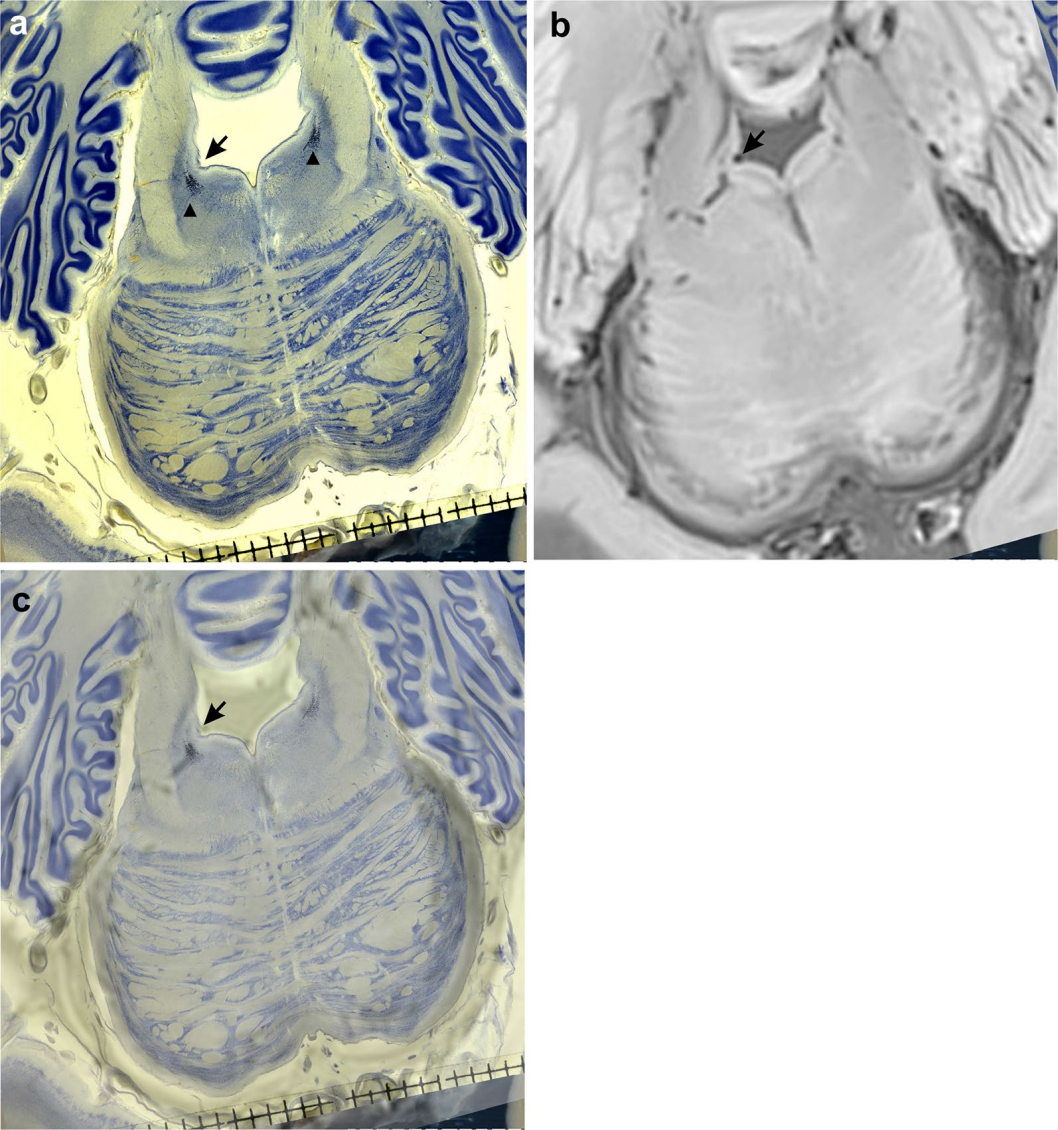
**a** Celloidin-embedded gallocyanin-stained 420-μm-thick horizontal brainstem section of a 85-year-old female case. **b** Axial 7 T post-mortem MRI (Siemens) after 3 months of formalin fixation of the complete brain—with a 3D gradient-echo acquisition (TE 8 ms, TR 12 ms, 0.6 mm thickness, 0.3 mm in plane resolution)—at corresponding horizontal level. **c** Merged **a** and **b** with semitrans parent **c** on the bottom of the stack. Arrows in **a** and **c** point to the lateral angle of the aqueduct. Arrowheads in **a** point to the locus coeruleus. Scalebar in **a** and **c** subdivided into mm

Finally, the paraffin- or formalin-embedded brain must be cut on special microtomes, that can carry 3–5 kg (weight of paraffin block + brains with cerebellum and brainstem) without vibration and gross variations in sections thickness with a range between 20 and 420 μm. It is necessary to CT scan the brain’s position (Figs. 2, 3, 4) in the block and to orient the brain with a horizontal line connecting the anterior and posterior commissure in a medio-sagittal plane. Cutting of brains causes additional deformations.

These strategies are time-consuming, but it is important that they are followed correctly. Shrinkage, swelling, and distortion artefacts in thick section (< 400 μm) are less frequently observed than in thin optical ones (≤ 20 μm) [13]. The superposition of several layers of neurons (more than 20 in a 420-μm-thick section compared with conventional 20-μm-thick sections) in the *z*-axis enhances minimal differences in size, shape, and staining properties of neurons. Cortical archtectonic borders and parcellation of subregions within subcortical nuclei are easier to be detected in thick sections after initial expert training. It remains to be tested to what extent visual cortical and subcortical parcellation are comparable with the so-called observer-independent computer-assisted parcellation [14]. Finally, the co-registration (or matching) process of MR images and thick histological sections need less corrections after carefully executed technical procedures.

Close-up macroscopic (Fig. 2b) and microscopic (Fig. 2c) photographs from gallocyanin-stained sections prove the superior resolution of Nissl-stained thick sections and the possibility to compare closely aligned 7 T images (Fig. 2a) with their histological counterpart. In addition, when assessed by means of a binocular-loupe, the transparent thick gallocyanin-sections provide a 3-D impression of human or animal brain architecture from neurons arranged in layers (Fig. 2b) at low magnification to individual cells flooded by erythrocytes at higher magnification (Fig. 2c).

Figure 2b demonstrates the impact of an aneurysm onto the entorhinal regions. The extended vascular wall caused a long-lasting indentation of the perirhinal (transentorhinal cortex) with considerable thinning of layers and probable neuron loss (reduced staining—pallor—with gallocyanin). The transentorhinal region is the first cortical region to be affected by tau pathology in the course of Alzheimer's disease [15]. It is considered a relay station between reciprocal iso- and allocortical information flow. The ruptured part of the aneurysm caused a small fountain of blood to spill erythrocytes into the medial entorhinal cortex. Phagocytosis of erythrocytes by macrophages and degradation of the red blood cells were just beginning to start. This is evidence that the time between rupture and death of the person differed by maximally two days, whereas neuron loss and distortion of layers in the perirhinal region persisted for a far longer time. The relatives of the diseased person did not report memory deficits. Unilateral pathology and/or missing sophisticated neuropsychological testing could explain an absence of reported neurological deficits. On the other hand, imaging and post-mortem histopathology could be a powerful tool correlating brain-structure interactions after selecting and diagnosing cases with circumscribed brain lesions that are associated with well-defined neurological or neuropsychological deficits. It is worth noting that human brain function and their morphological correlations are based on comprehensive careful case reports from as far back as the start of the past century, or even earlier.

Figure 3a depicts sharp borders of the basal ganglia and the thalamus, whereas Fig. 3b shows CSF-filled perivascular spaces (extended Virchow–Robin spaces) and a small infarct in caudal (posterior) thalamic regions. If these widened perivascular spaces are dispersed over the striatum, they can be recognized with the unaided eye (diameter < 5 mm) or in a low magnification—neuropathologists classify these changes as status cribrosus. In our case, we can observe the outlines of the globus pallidus between the crura of the capsula interna medially and the putamen laterally. The outlines of both globi pallidi are better to be seen in Fig. 3a. The globus pallidus on the left side is stained in a faint purple hue, on the right side in a yellow to brown one. This is an indication of either a high concentration of iron ions, lipofuscin or both.

These tissue changes indicate neuronal degeneration and extraneuronal accumulation of iron, which normally plays an important role as a catalyser in enzymatic processes. At higher magnification, the borders of the thalamic ventrocaudal nucleus and the pulvinar thalami can be unequivocally seen. Parcellation of thalamic subnuclei in conventional 20-μm-thick paraffin sections can be a matter of dispute, even between experts in this field [16]. The old thalamic infarct is surrounded by a lightly stained capsule that consists of fibrouas astroglial cells forming a neuron-free scar. However, in the periphery of the infarct, single, viable, as well as irregularly shaped groups of intact and degenerating neurons can be identified. This kind of neuropathological degeneration is known as elective parenchymal necrosis. It will result in complete neuron loss and replacement of neurons, as well as their processes, by an astroglial scar and the absence of a fluid-filled cyst. The less conspicuous sickle-shaped hyperintensity below the central cyst in Fig. 3d is most likely the 7 T equivalent of this still ongoing process. In addition, widened perivascular spaces around arteries supplying the caudate nucleus and pulvinar (status cribrosus) indicate a leakage of the blood-brain-barrier and disturbed blood supply in both striata of this case [29–33].

## The potential of high-resolution 7 T MR scanners in analysing regions with early Alzheimer’s disease (AD) related pathology

The locus coeruleus forms an elongated slender rostrocaudally oriented column in the brainstem consisting of conspicuous predominantly pigmented neuromelanin containing neurons (Fig. 4a–c). The axons of these neurons are thin. However, they leave the brainstem in a rostral direction to the telencephalon and in a caudal course to the spinal cord. After a distance of several centimetres, the axons collateralize and a maximal unilateral number of 137,910 (58-year-old Braak stage II case) pigmented neurons will supply billions of telencephalic cerebellar, and spinal cord neurons with noradrenaline [34]. Noradrenaline is considered a neuromodulator that enhances vigilance and attention.

In three Braak stage VI AD cases, ranging in age from 78 to 81 years, the locus coeruleus was devoid of neurons [34]. According to these post-mortem quantitative analyses, locus coeruleus volume already declines in the earliest AD stages (stages a-c according to Braaks’ modified classification [35, 36]) and after a time interval in addition to volume decrease, neurons start dying (Braak and Braak stage II). The rostral and the middle parts of the locus coeruleus show earlier volume and more neuron loss than the caudal one. Neuronal death in AD signifies a point of no return in AD progression because neurogenesis in the adult human brain is absent or, if at all, confined to the hippocampal dentate gyrus.

Recently, the locus coeruleus could be visualized with 3 T imaging [37]. A combined 7 T post-mortem in situ volumetric study, supplemented by a post-mortem stereological investigation on serial gallocyanin and hyperphosphorylated tau immunohistochemically stained sections of the same brains, could answer the question as to whether longitudinal voxel-based morphometric studies are a replicable and reliable marker of AD progression. The locus coeruleus would be ideal for this kind of study, because to our knowledge, locus coeruleus neurons are prone to early AD-related pathology. This is obviously long before cortical regions are affected and, consequently, patients become demented and are subject to long-lasting intensive and expensive care.

The nucleus basalis of Meynert (NbM) forms an integral part of the human substantia innominata and it supplies the telencephalic iso- and allocortex with acetylcholine [38, 39]. Cortical decrease in acetylcholine is an early neurochemical marker of AD [40, 41]. Therefore, it would be an additional candidate for a combined post-mortem in situ 7 T histological study. NbM neurons show tau pathology already in Braak stages 0 and I, similar to locus coeruleus neurons [42]. Its neurons are arranged in complex curving band coursing in the basal forebrain [43]. After mapping basal forebrain cholinergic nuclei into the MRI standard space, signal changes could be correlated with grey matter atrophy and other parameters [44–54].

Presently, it is not possible to visualize the NbM with 3 T or 7 T imaging. In similar protocols like the proposed test of the feasibility of locus coeruleus morphometry, the stereological analysis of total NbM neuron number and nuclear volume could mark atrophic changes (loss of dendrites) and decreasing perikaryal (Nissl-stained cytoplasm surrounding the characteristic neuronal nucleus + nucleolus) from necrotic irreversible neuron loss. The resolution of 7 T scanners is still too low to directly address the questions of cellular atrophy, cell death, reactive astrogliosis, microglial reaction and loss of oligodendrocytes (Figs. 2, 4 document this). A recent series of publications summarizes this scanner-associated resolution deficit under the term microstructure and several strategies suggest to bridge this shortcoming [55–58]. Likewise, tractography studies should be supplemented by histological protocols [59].

## The role of computer science, AI and ML in integrating virtual reality (MR imaging) with histological reality (documentation of histological slides with digital cameras or scanners)

The relatively low resolution of 7 T MR scanners compared with high power resolution of advanced digital cameras, scanners, and microscopes suggests a combination of both methods. In vivo and selected post-mortem in situ 7 T scans provides images of an intact brain more or less free from manipulation-associated artefacts. The increase in resolution of histologically processed brains comes at a price of processing artefacts, starting with fixation, and ending with coverslipping of the individual stained sections.

Depending on the plane of section (coronal, horizontal, or sagittal), a stack of around 330–280 serial sections with a section thickness of 400–420 μm must be photographed with high-resolution digital cameras during the section procedure with a heavy duty microtome. High-resolution colour photographs taken through the central parts of the brain can comprise up to 15 MB in JPG format or up to 80 MB in the TIF-format. Blockface pictures of serially cut brains provide a template for arrangement and stacking of the sections that are stained after the cutting procedure.

The next step includes the co-registration of the stacked and coloured sections into the MRI space. The high-resolution pictures yield a high number of data that must be processed with supercomputers [60]. This procedure provides high-resolution virtual slices that can simulate planes of sections other than the ones originally produced on the microtome. This greatly facilitates cytoarchitectonic delineations and localization of small circumscribed MRI structures or profiles of uncertain nature and origin in the MRI [61].

The following step would be the stereologic analysis of cell density, cell size, and cell type (glia, neurons). This is an extremely time-consuming procedure, because the investigator must unequivocally identify typical features of glial cells and neurons whilst focusing on the z-axis of a microscope. Superimposition of densely packed cells complicates this task. We could automate the analysis in immunostained 60 μm sections [62]. With this procedure, cells and profiles are well-defined and easier to classify. Nissl-stained sections impose far more challenges in classifying cellular profiles and Schmitz et al. concluded that present automated methods are unreliable [63].

Voxel-based morphometric imaging with 3 T or 7 T scanners clearly demonstrates volume loss or density changes in the course of neurodegenerative diseases. However, presently, the underlying causes for these changes can only be disclosed by a combined MRI-histological analysis of the affected brain. Stereology provides a number of mathematical methods and guidelines that guarantee replicable results with analysis of histological sections [64]. The disector guidelines [65, 66] prevent overestimation of cell number when analysing cells of different shape and size. Schmitz and Hof provide a comprehensive overview for analysis of other neuronal elements besides perikarya [67]. Cell number estimation with stereological tools and disector principles represent a special case of particle analysis in a 3D space. A human researcher must identify profiles when focusing within a defined optical thickness and count all neurons or glial cells that arise in his/her microscopic field of vision. Consequently, an automated computer-based strategy must coordinate pattern recognition with defined movements within a 3D space. Pattern recognition is presently the most intriguing step, and to our knowledge, no unanimously accepted strategy is available. This technique would greatly reduce the cumbersome burden of cell counting, and a combination of both methods (as we have described) will enhance the diagnostic and prognostic capabilities of 3 T or 7 T scanners by providing biomarkers.

Another remaining open question is the strategy of data processing. Amunts et al. [69] studied paraffin-embedded brains, which were cut into more than 7000 20-μm-thick slices. They were using only every 20th section for data acquisition and they concluded that a comprehensive analysis of their data exceeds present computer capabilities [68, 69]. We have profited by the Berkeley supercomputer facilities for co-registration of our high-resolution macroscopic pictures of a human brain [60]. We estimate that high-resolution microscopic pictures of our thick serial slices would exceed the data provided by Amunts et al. by a factor of 10.

Furthermore, already routine diagnostic assessment of 3 T or 7 T images yields an immense body of biographical and clinical data of patients. Molecular imaging protocols provide additional aspects and data [70–74]. In animal research, mean diffusivity changes could be related to structural changes in astrocytes, myelin or synaptic remodelling [75, 76]. Taken together, this immense conglomerate of data warrants advanced data processing and interpretation.

For successful ML, we need the combination of data from various sources. Here we must mention that for automatic ML we need a substantial amount of data sets (“Big data”). Particularly deep neural networks require enormous amounts of training sets until an algorithm can reliably identify patterns and structures within a brain image. Since it is often difficult to get millions of brain images, there are some image augmentation methods available, which can help to produce the necessary amount of training data. However, two crucial facts are always underestimated in the medical domain: (1) we need not only top-quality data, but (2) we need domain expertise. Ultimately, the results have to be validated. We have to emphasize that ML does not mean that the software learns what, e.g. “the hippocampus is and how it looks”. Rather, the algorithm only learns (biologically meaningless) patterns within images, and those patterns can then be identified via image-processing methods. This is important because this actually causes limitations in machine-based identification of brain regions and pathologies. Consequently, providing high-resolution images in general, and MRI data in particular, not only supports the development of ML algorithms for image analysis per se, but enables us to reach a further step towards diagnosis assistance [77, 78].

Whilst AI methods have shown to be equally or even more effective than human clinicians in diagnosing dementia from neuroimages [79], further clinical studies are needed (beyond what is presented in this paper), which should include full cases to provide important findings based on histological (e.g. thin slices of whole brain) and MRI data (e.g. in situ, ex-situ) [80, 81]. So far, psychotic diseases cannot be diagnosed histologically. Investigations into psychiatric diseases with unknown histological markers could also be beneficial. We hypothesize that AI could point out to which areas might have differences in these populations, and each brain could be histologically assessed. This is not yet possible with current in vivo studies, but this assessment could be expanded if more tissues were made available for histological analysis.

Our group has been using automatic classifiers, mainly support vector machines (SVM) to analyse both structural and functional MRI datasets in patients and healthy volunteers [82–88]. The majority of the findings in neurodegenerative diseases point to a reduction in brain morphometrics—conversely, patterns related to cortical malformations are known to produce cortical thickening (in fact, when analysed closely, this macroscopic appearance is due to intracortical disorganization of the layers in the neocortex). Changes that are more subtle—and today still remain undetected by experts—can be unravelled using texture analysis methods combined with classifiers [88]. We believe that those features will benefit from the use of ultra-high field MR data and recent developments in ML technologies. Disease classifications based on ML-methods, that incorporate data from studies using chemical imaging will expand the scope of possibilities. This idea of quantitative mapping incorporates data from particular chemical components at spatial and temporal resolution [89].

For classification of nuclei, Sirinukunwattana et al. used Neighbouring Ensemble Predictors [90] coupled with (meanwhile very successful) standard convolutional neural networks [91] to more accurately predict class labels of the detected cell nuclei, which do not require segmentation. Such methods can offer huge benefits to pathology practice in terms of quantitative analysis of tissue constituents in whole-slide images, and potentially lead to a better disease understanding. This can be further enhanced by bringing a “pathologist in the loop”, i.e. by application of interactive ML (iML) methods [92, 93], which also enable to explain *why* a machine decision has been reached [94, 95]. Technically, this will be done by applying algorithms which enable an integration of a human into the algorithmic loop, who can then provide his/her expertise to find the underlying explanatory factors [96].

## Conclusion

In an analogy with architecture, enormous constructions should be based on a solid foundation. The cooperation of disciplines including neuroimaging and neuroanatomy/neuropathology could provide a fundament of solid and replicable data. Presently, virtual autopsy cannot substitute conventional autopsy. Both methods should be used concomitantly with provided tissue and organs for a comprehensive and meticulous research database for control and diseased cases.

## Abbreviations

AD: Alzheimer’s disease
AI: artificial intelligence
CNN: convolutional neural network
CT: computerized tomography
FLAIR: fluid-attenuated inversion recovery
GRE: gradient echo
HD: Huntington’s disease
LDA: linear discriminant analysis
ML: machine learning
MR: magnetic resonance
MRI: magnetic resonance imaging
MRS: magnetic resonance spectroscopy
PD: Parkinson’s disease
TE: echo time pulse sequence parameter
TR: repetition pulse sequence parameter
T1: spin–lattice weighted image relaxation time
T2: transverse weighted image relaxation time
Virtopsy: virtual autopsy
USP: University of Sao Paulo
3 T: 3 Tesla
7 T: 7 Tesla
